# An analysis of the foot in turnout using a dance specific 3D multi-segment foot model

**DOI:** 10.1186/s13047-019-0318-1

**Published:** 2019-02-04

**Authors:** Sarah L. Carter, Alan R. Bryant, Luke S. Hopper

**Affiliations:** 1 Podiatric Medicine and Surgery Division, School of Allied Health, The University of Western Australia, Perth, Australia; 2 Western Australian Academy of Performing Arts, Edith Cowan University, Perth, Australia; 3 Podiatric Medicine and Surgery Division, M422, 35 Stirling Highway, Crawley, WA 6009 Australia

**Keywords:** Ballet, Three-dimensional, Hallux valgus, Kinematics, Sautés

## Abstract

**Introduction:**

Recent three-dimensional (3D) kinematic research has revealed foot abduction is the strongest predictor of standing functional and forced turnout postures. However, it is still unknown how the internal foot joints enable a large degree of foot abduction in turnout. The primary purpose of this study was to use a dance specific multi-segment foot model to determine the lower leg and foot contributions to turnout that female university-level ballets use to accentuate their turnout.

**Methods:**

Eighteen female dance students (mean age, 18.8 ± 1.6 years) volunteered for this study. Retro-reflective markers were attached to the dancers’ dominant foot. Each dancer performed three repetitions of functional turnout, forced turnout and ten consecutive sautés in first position. Repeated measures ANOVA with Bonferroni adjustments for the multiple comparisons were used to determine the kinematic adjustments, hindfoot eversion, midfoot and forefoot abduction, navicular drop (i.e. lowering of the medial longitudinal arch) and first metatarsophalangeal joint abduction between natural double leg up-right posture and the first position conditions.

**Results:**

Hindfoot eversion (4.6°, *p* < 0.001) and midfoot abduction (2.8°, *p* < 0.001) significantly increased in functional turnout compared to the natural double leg up-right posture. Thirteen dancers demonstrated increased first metatarsophalangeal joint (MTPJ) abduction in forced turnout, however no statistically significant increase was found. Navicular drop during sautés in first position significantly increased by 11 mm (*p* < 0.001) compared to the natural double leg up-right posture.

**Conclusion:**

Our findings suggest dancers do pronate, via hindfoot eversion and midfoot abduction in both functional and forced turnout, however, no immediate association was found between forced turnout and first MTPJ abduction. Foot pronation does play a role in achieving turnout. Further prospective research on in situ measures of the lower limb in turnout and injury surveillance is required to improve our understanding of the normal and abnormal dance biomechanics.

**Electronic supplementary material:**

The online version of this article (10.1186/s13047-019-0318-1) contains supplementary material, which is available to authorized users.

## Background

A dancer’s foot articulates through extreme ranges of motion to achieve a wide variety of aesthetically pleasing dance movements and postures. Our understanding of the internal movements of the foot joints in dance is largely based on theoretical knowledge, in particular, the compensation mechanisms the foot undergoes to achieve a greater turnout due to limited hip external rotation [1, 2]. Turnout involves the maximal external rotation of the lower limb and is fundamental to classical ballet. 3D motion analysis is a growing field in dance research, which is enhancing our understanding of dynamic alignment during the execution of specific dance movements. Previous 3D kinematic research has revealed foot abduction is the strongest predictor of a dancers’ turnout in first position [3]. However, it is still unknown how the internal foot joints enable a large degree of foot abduction in turnout.

**Fig. 1:**
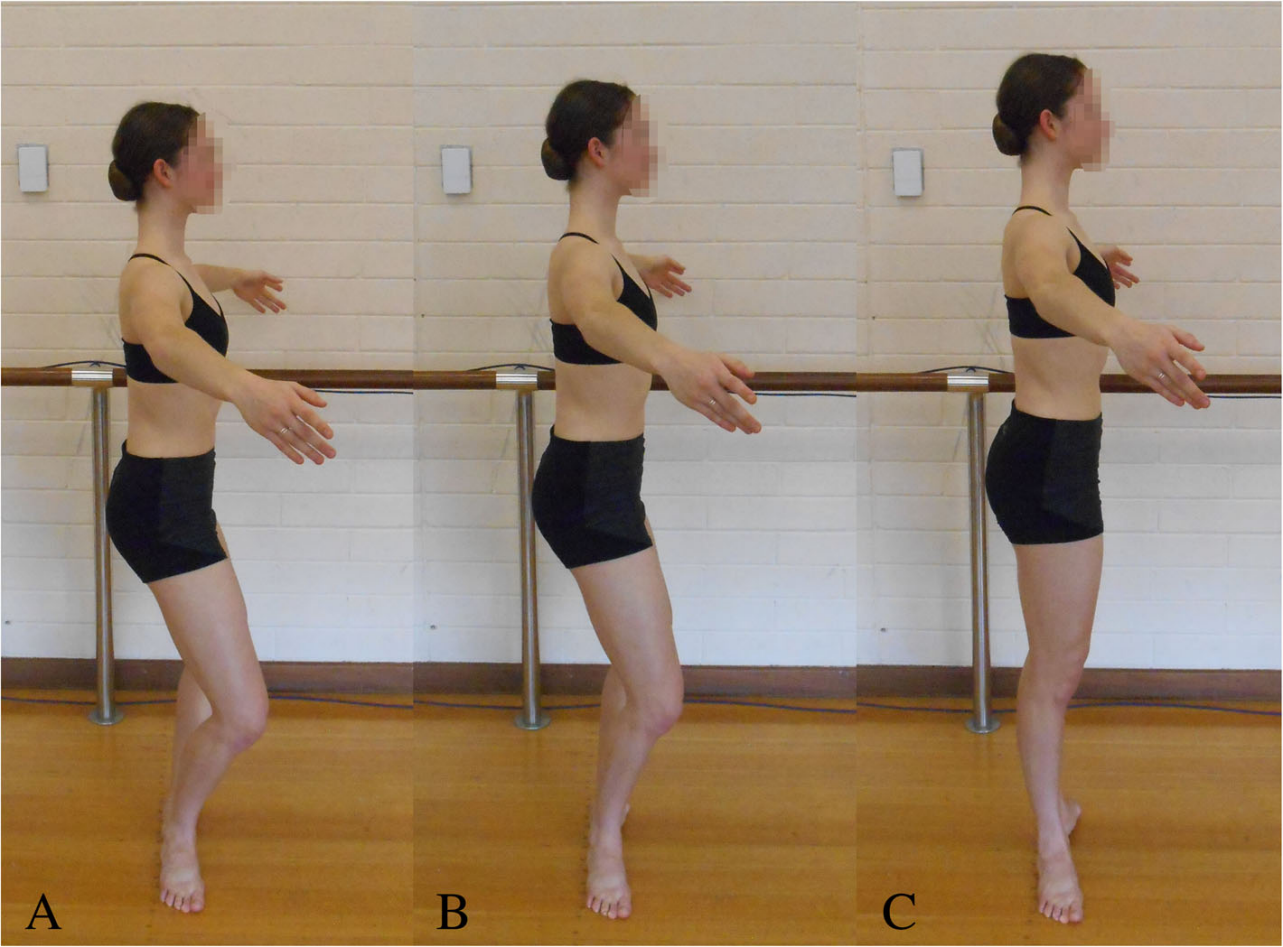
Sagittal view of a dancer purposely demonstrating forced turnout in first position. Dancers usually position their feet in an exaggerated turnout angle in demi-plie (**a**), a dancer maintains this angle while extending through the knees (**b**), into a forced turnout double leg up-right posture in first position (**c**). The dancer is also demonstrating poor knee-foot alignment (**c**)

**Fig. 2:**
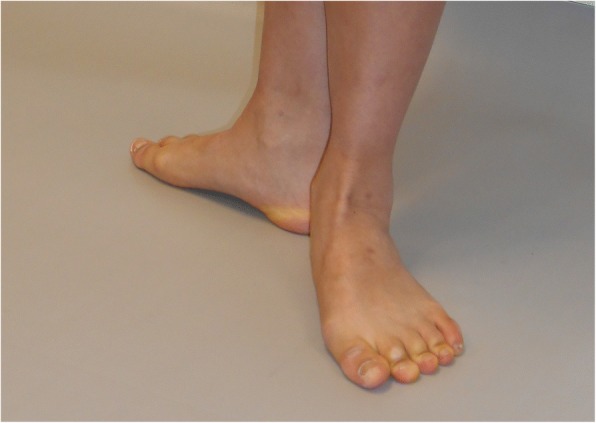
Hyper-pronation of the foot in forced turnout. Dancer demonstrating hindfoot eversion, medial bulging of the talar-navicular joint, forefoot abduction, toes gripping the floor and lateral deviation of the hallux

Dancers will often assume a comfortable double leg up-right posture in turnout in class and this is commonly referred to as their ‘functional turnout angle’. First position in turnout is with both hips maximally externally rotated, the knees are extended with the heels contacting, the knee should be in line with the second metatarsal, and the longitudinal axes of the feet pointing away from each other [4]. Dancers with low hip external rotation are likely to compensate through foot abduction, a component of pronation, rather than forcing turnout via knee external rotation [3]. Dancers will often force turnout by planting their feet in an overturned position in demi-plié (knee bend) (Fig. 1A) followed by knee extension (Fig. 1B-C). The foot maintains a passive overturned position via the friction between the plantar surface of foot and floor. The rotational force created from the floor is thought to be attenuated via the tibia internally rotating relative to the foot [5] and subtalar joint pronation (i.e. eversion of the calcaneus, plantar flexion and adduction of the talus) [5, 6], resulting in the midtarsal joint unlocking, allowing forefoot abduction [2, 6, 7], and lowering of the medial longitudinal arch [1, 2, 8] (Fig. 2).

A dancer’s foot posture in turnout forms an important part in dance screening programs [9–11]. Research has demonstrated that dancers attain a more pronated posture [6, 12] (i.e. a greater Foot Posture Index-6 score [13]) and a lower medial longitudinal arch profile [12] (i.e. navicular drop [14]) in functional turnout compared to their natural double leg up-right posture. Conversely, dance research using 3D kinematic analysis, with a retro-reflective marker attached to the navicular tuberosity [15], reported no significant change in the medial longitudinal arch height stability for both a dancer’s functional turnout and forced turnout position [3]. These findings disagree with the common anecdote that the medial longitudinal arch lowers in forced turnout [1, 2, 8]. However, Carter et al. 2018 [3] did not consider the dancers’ foot type, it is unknown whether any of the dancers were highly pronated (10+ Foot Posture Index-6 score) or highly supinated (− 5 to − 12 Foot Posture Index-6 score) [13]. The following point that can be identified from the previous published work on turnout, is that dancer’s use foot pronation as a compensation strategy to achieve a greater turnout and, yet the internal mechanisms used for pronation by dancers are still ill-defined.

The hindfoot provides the mechanical connection between the foot and the lower limb in which rotational movements of the lower leg are translated into frontal plane movements of the foot [1]. In walking, foot abduction is achieved via the closed kinetic chain axial movement of the tibia which produces eversion of the hindfoot and abduction of the forefoot, a transverse plane motion [1, 16]. Dysfunction in the coupling of this intersegmental relationship, has already been investigated clinically [12] and using 3D motion analysis [3]. Dancers with a low pronated (abducted) position in functional turnout, forced turnout and during sautés (i.e. a small jump task which begins and finishes in a plié) demonstrated high passive external tibiofemoral rotation [3]. This finding suggests that limited hindfoot eversion is compensated by increased passive external tibiofemoral rotation in turnout. This is consistent with current biomechanical theory where a hypo mobile joint is compensated or transferred to the next mobile joint along the closed kinetic chain [1]. As earlier discussed, dancers are more likely to compensate for limited hip external rotation via foot pronation, followed next by knee external rotation in spite of the knowledge that poor turnout technique is associated with a high risk of injury [17–20].

A hyper pronated foot in turnout is associated with increased mechanical stress and loading on the following structures; plantar fascia [1, 2, 21], deltoid ligament [1, 8], tibialis posterior muscle [1, 21, 22] and the gastro-soleus complex [2, 8, 21, 23–25] and the first metatarsophalangeal joint (MTPJ). Quantitative measurement of in situ 3D segmental movements of the foot using a dance-specific modified Rizzoli Foot Model [26–28] during turnout may assist in understanding the risk factors for these foot related injuries and stressors in dancers.

The condition of hallux valgus (bunions) has anecdotally been associated with ballet participation, even though there is conflicting evidence whether dancing increases the risk of developing hallux valgus deformity [29–32]. Hallux valgus is a deformity where the hallux or great toe is laterally deviated more than 15° from the bisection of the first metatarsal [33]. A ballet-related biomechanical aetiology for hallux valgus has been proposed in which forcing turnout gradually leads to stretching of the interosseous ligaments between the first and second metatarsal heads, allowing hallux abduction and a greater valgus force on the first MTPJ [1, 30]. More recently, research is suggesting an underlying genetic predisposition in conjunction with excessive mechanical stress on the growth plates of young dancers may explain an increased incidence of hallux valgus observed in the dancing population [29]. Application of a dance-specific modified Rizzoli foot model will enable dynamic assessment of the first MTPJ transverse plane position, which can assist in investigating the biomechanical aetiology around hallux valgus.

In vivo evaluation of dancers’ leg and foot posture can enhance our understanding of the mechanical adjustments dancers utilise to further increase their turnout and the associated tissues at risk of mechanical stress. Therefore, the primary purpose of this study was to use a dance-specific 3D multi-segment foot model to determine the lower leg and foot contributions to turnout that female university-level ballets use to accentuate their turnout. We hypothesised that 1. A dancer’s foot will undergo hindfoot eversion, lowering of the medial longitudinal arch, midfoot and forefoot abduction in forced turnout compared to functional turnout. 2. A dancer’s hallux will be significantly more abducted in forced turnout compared to their natural double leg up-right posture. 3. The hindfoot eversion is the main contributor of foot abduction during first position conditions. 4. Passive external tibiofemoral rotation will negatively correlate to hindfoot eversion during first position conditions.

## Method

### Study design

This study used a cross-sectional study design.

### Selection and description of participants

Eighteen dance students (fourteen classical ballet and four modern dance) (mean ± SD: age, 18.8 ± 1.6 years, height, 1.66 ± 0.7 m, body mass, 58.3 ± 5.3 kg, Foot Posture Index-6, 1.3 ± 2.8) from the Western Australian Academy of Performing Arts volunteered for this study. Dancers were excluded if they were currently injured and/or they had highly pronated foot (10+ Foot Posture Index-6 score) or highly supinated foot (− 5 to − 12 Foot Posture Index-6 score) [13]. Descriptive statistics for the participants dance history are summarised in Table 1.

**Table 1:** Dance history characteristics of university-level ballet dancers (*N* = 18)

Characteristic	Mean ± SD	Range
Age started dancing (yrs.)	6.2 ± 3.4	2–13
Years of ballet training (yrs.)	12.6 ± 3.6	5–17
Ballet training (hr/wk.)	19.5 ± 8.8	1.5–35
Dancing barefoot (hr/wk.)	12.1 ± 8.1	4–30
Rehearsals (hr/wk.)	8.2 ± 4.7	0–18
Technique (hr/wk.)	12.7 ± 5.8	4–27.5
Stretching (hr/wk.)	3.6 ± 2.1	0–8

All 18 participants signed an informed consent form. Ethic approval was obtained by the Edith Cowan University Research Ethics Committee (12,534 HOPPER).

### Data collection

**Fig. 3:**
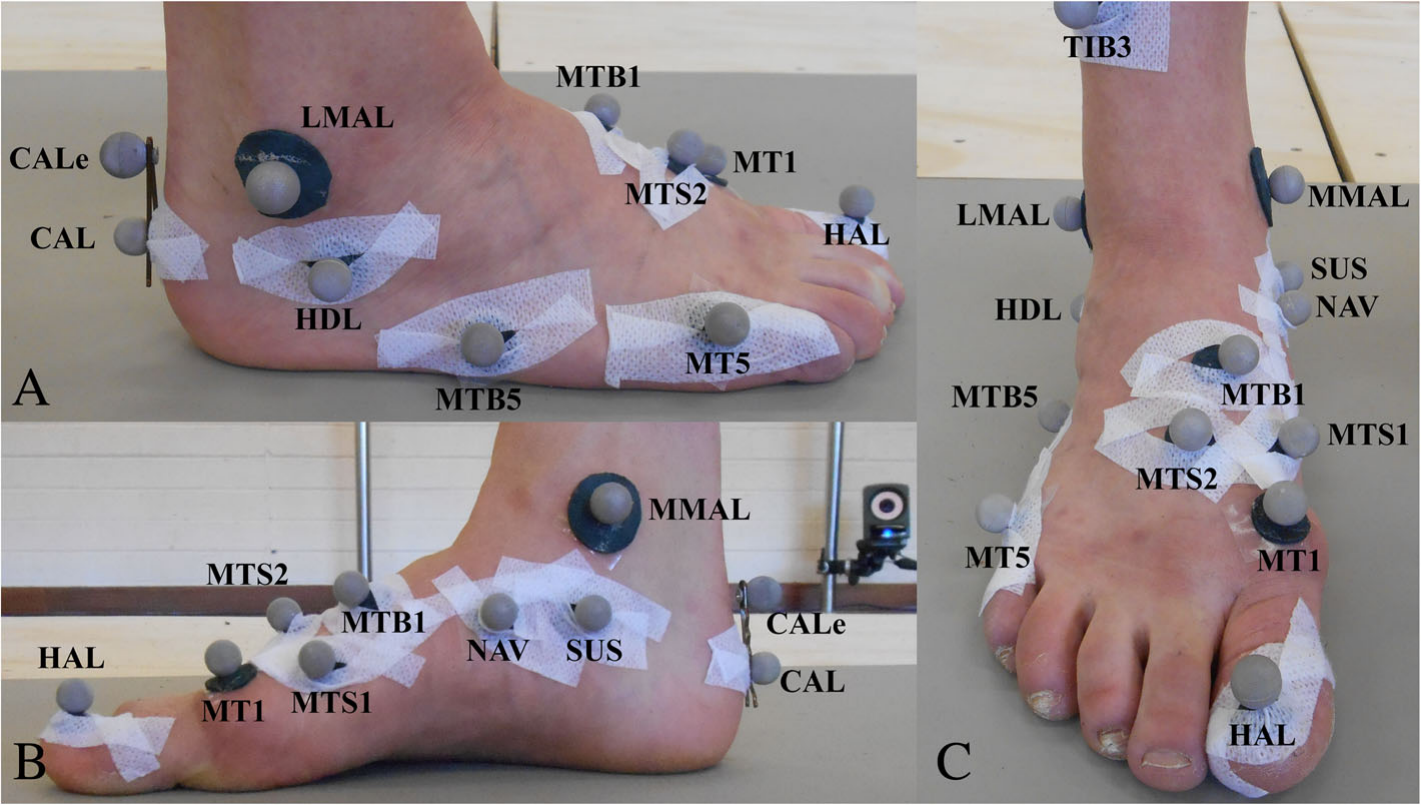
Lateral view of the marker placement (**a**) medial view of the marker placement (**b**) and anterior view of the marker placement (**c**)

Limb dominance was determined using the Waterloo Footedness Questionnaire [34]. Retro-reflective markers were attached to the dancers’ dominant lower limb and foot in accordance with the positions described in the Besier, Sturnieks, Alderson, & Lloyd (2003) [35] lower limb model and a dance-specific modified Rizzoli Foot Model [26–28] (Fig. 3). The inter- and intra-assessor repeatability and modifications of the multi-segment model was previously established on six female pre-professional dancers [28]. Twelve, Vicon T40S cameras and one Bonita video camera (Oxford Metrics, UK) were calibrated to track the individual retro-reflective markers and triad marker clusters on the pelvis, thigh, lower leg, foot, hindfoot, midfoot, forefoot and hallux at 250 Hz with an error of < 1 mm. A natural double leg up-right posture trial captured the anatomical calibration marker locations relative to technical reference frames; lateral and medial malleolus relative to the tibia marker triad, MT1 relative to a MTS1, MTS2 and MTB1 and CALe relative to the hindfoot markers (CAL, SUS, and HDL) [28]. A standing lunge trial recorded the femoral condyle marker positions held to thigh marker triad, with the mid-point of the condyle markers used to create the knee joint centre. The knee symmetrical axis of rotation (SARA) [36] was determined by the dancers performing 4 consecutive squats, followed by moving the hip joint through forward flexion, abduction, backward extension and circumduction in an externally rotated position to calculate the hip symmetrical centre of rotation estimation (SCoRE) [37]. These were performed in order to calculate knee external rotation (i.e. external tibiofemoral rotation). All participants performed a standardised 10 min ballet specific warm-up routine before data collection commenced.

**Fig. 4:**
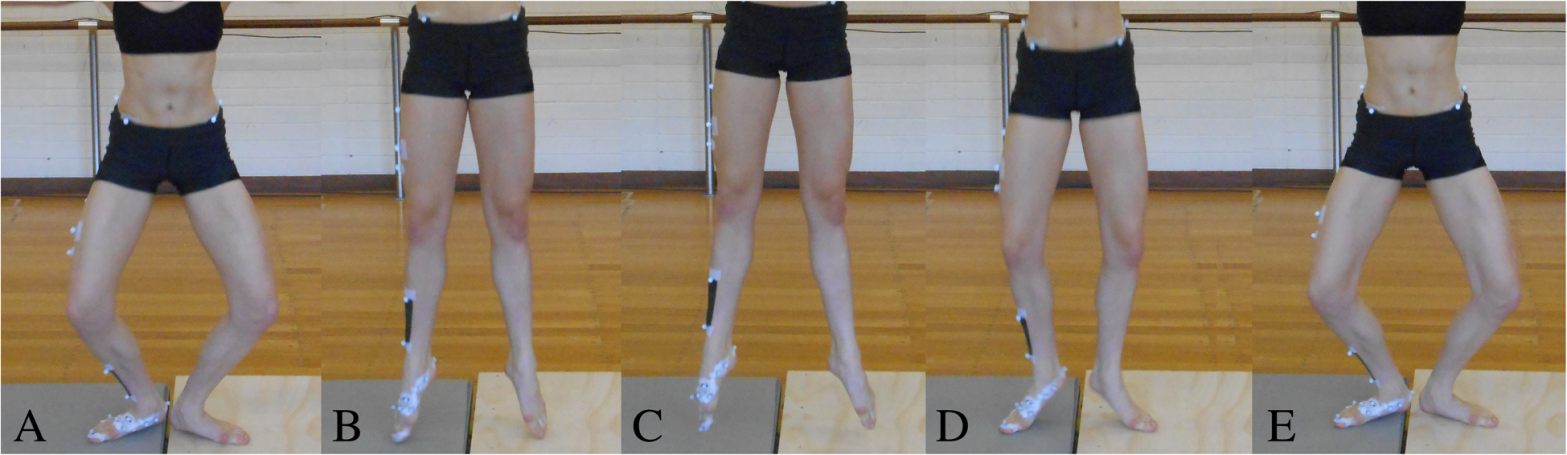
Frontal view of a dancer performing a sauté in a slightly open first position. A sauté begins from a demi-plié position (**a**) the sauté movement is initiated by the simultaneous extension of the hips and knees and plantar flexion of the ankles. This shifts the body weight from the heel to the forefoot, as the dancer moves onto demi-pointe [55] (**b**). The last point of contact before elevation is with the toes [55]. In the air, dancers maximally plantar flex the whole foot (pointe) (**c**). Dancers are taught to land with a nearly fully extended knee and a maximally plantar flexed foot at initial contact [55, 56]. The phalanges are the first point of contact, followed by ‘rolling through their feet’ to allow the heels to contact the ground quietly [56] (**d**). The sauté ends with a demi-plié (**e**)

Each dancer then performed three first position conditions:

1. **Functional turnout**: Participants were instructed to stand still in their preferred turnout angle in a slightly open first position (heels not in contact), so not to occlude the hindfoot markers. This was repeated three times.
2. **Forced turnout:** Participants were instructed to stand still in a forced turnout angle in a slightly open first position. Dancers had to be able to maintain balance while holding the forced turnout angle, if they were unable to maintain balance, the dancer was informed to slightly reduce the angle until balance was maintained during the trial. This was repeated three times.
3. **Sautés** (double leg small jump): Participants were instructed to perform ten consecutive sautés in a slightly open first position (Fig. 4) at a controlled tempo of 95 beats per minute.

Passive and active external tibiofemoral rotation measurements were recorded in a seated position with the hip in neutral relative to the frontal and transverse plane. Passive external rotation was performed with the examiner manually rotating the lower leg, while stabilising the thigh. When the point of resistance was felt, the foot was placed on the ground. Femoral epicondyle markers were reattached to redefine the knee joint centre and a single trial captured this position. Active external rotation was performed with the foot on a freely rotating disc to minimise friction. Dancers were instructed to actively externally rotate from below the knee while maintaining thigh position. A single trial was recorded with the dancer holding this externally rotated knee.

#### Data processing

**Fig. 5:**
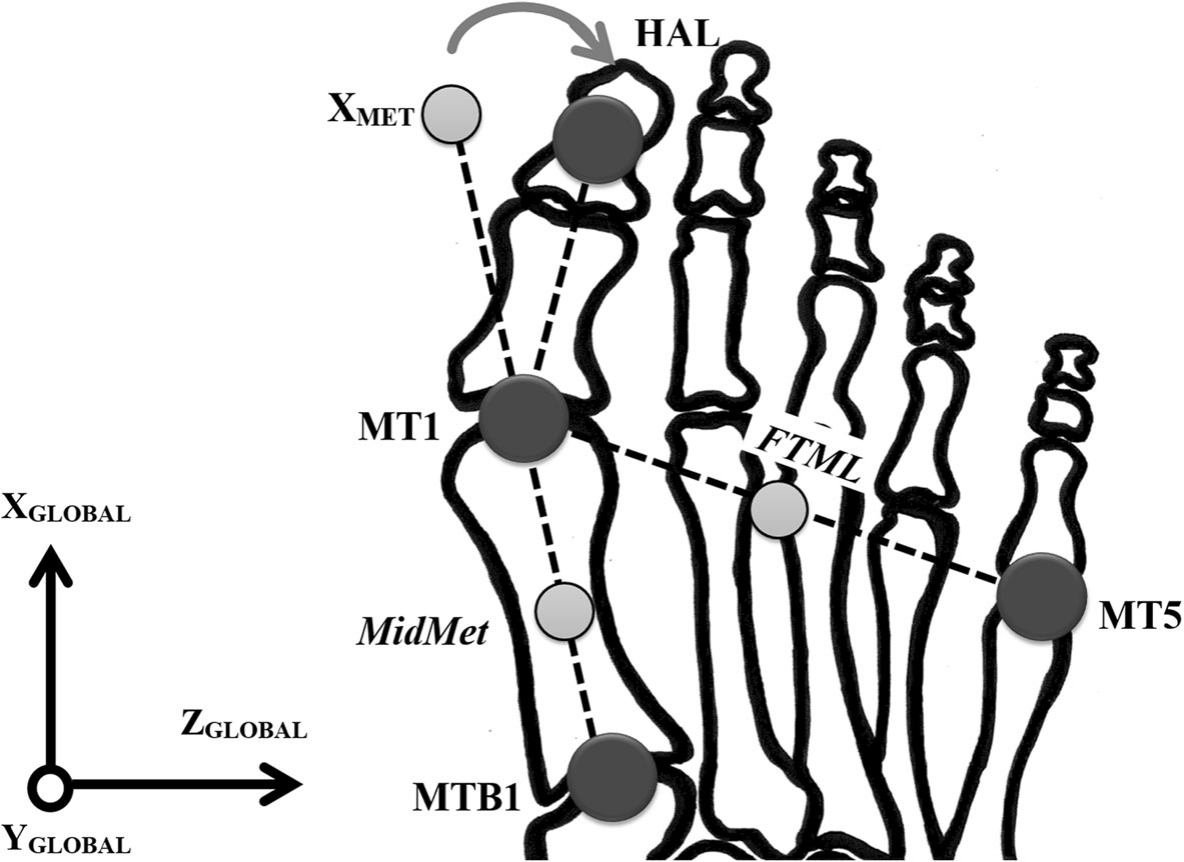
Diagram depicting an abducted first MTPJ in the transverse plane. The dark grey circles represent the anatomical retro-reflective markers and the light grey circles represent the calculated midpoints. Note: MTB1, base of the first metatarsal (anatomical/tracking marker); MT1, head of first metatarsal (anatomical/calibration marker); *MidMet*, midpoint between MTB1 and MT1 (virtual marker); X_MET_, a virtual marker on the end of the x-axis defined by the vector joining the metatarsal origin (MT1) and MTB1, and pointing anteriorly; HAL, middle of the dorsal aspect of the hallux nail (anatomical/tracking marker); MT5, head of the fifth metatarsal (anatomical/tracking marker); *FTML*, intermedius forefoot, mid-point between MT1 and MT5 (virtual marker)

Raw 3D marker coordinates from the sauté movements were filtered using a low-pass Butterworth filter at a cut-off frequency of 26 Hz determined through a residual analysis [38]. Vicon Nexus 2.3 and Bodybuilder software (Oxford Metrics, UK.) were used in the reconstruction of the virtual markers in each trial (femoral epicondyles, malleoli (LMAL, MMAL), hindfoot offset (CALe), first metatarsal head (MT1)), using the Calibrated Anatomical System Technique method [39]. Joint coordinate systems were constructed for the hip, knee and ankle joints [35]. Joint rotations according to the International Society of Biomechanics Conventions [40, 41] were applied to the multi-segment model; movement of the hindfoot with respect to the tibia (TIB-HIND) i.e. the ankle/subtalar joint complex, the midfoot with respect to the hindfoot (HIND-MIDF) i.e. the midtarsal joint, the forefoot with respect to the midfoot (MID-FORE) and the entire foot in respect to the tibia (TIB-FOOT). The transverse planar movement of the hallux marker (HAL) was measured with respect to the transverse (X_MET_) vector of the first metatarsal (MET) segment (Fig. 5). The first MTPJ abduction angular measurements were classified as normal (< 15°), ‘mild’ (15–20°), ‘moderate’ (21–39°), and ‘severe’ (≥ 40°) in keeping with Piqué-Vidal & Vila [33]. The intra- and inter-assessor repeatability of the first MTPJ abduction angle was determined using legacy data from Carter et al. [28] (see Additional file 1). The intra- and inter-assessor values for first MTPJ transverse plane angles demonstrated excellent repeatability [42] with intra-class correlation coefficient values ranging between 0.886 and 0.888.

The lowering of the medial longitudinal arch was determined using the height of the marker on the navicular tuberosity, i.e. navicular drop [15]. Navicular height in relation to the laboratory coordinate system (in millimetres) was analysed with the variation in height from the natural double leg up-right posture indicating navicular drop. Navicular drop was classified into three types of arch height stability according to Gontijo et al. [43]: 1. ‘excellent stabilisation’ navicular drop < 7 mm); 2. ‘stable’ (navicular drop from 7 to 13 mm); 3. ‘unstable’ (navicular drop > 13 mm).

Data extraction and averaging of the 3D inter-segmental joint angles; tibia-foot transverse plane, tibia-hindfoot frontal plane, hindfoot-midfoot transverse plane, midfoot-forefoot transverse plane, first MTPJ transverse plane, knee transverse plane, and the height of the marker on the navicular tuberosity for each dancer was performed using MATLAB (custom software written using MATLAB, MathWorks Inc., USA). Three consecutive sautés with consistent pelvic height excursions were chosen for analysis. The kinematic variables mentioned above were extracted at the time the pelvis reached the lowest vertical height, the point when a dancer is in demi-plié. An average of the three demi-pliés for each kinematic variable was then calculated. Mean values for the measured variables across the repeated static trials of functional turnout and forced turnout were calculated. For the single static trials: natural double leg up-right posture, passive external tibiofemoral rotation and active external tibiofemoral rotation, a single value was calculated for each measured variable by determining the mean across the trial.

### Statistical analysis

All data were analysed using the Statistical Packages for Social Science (SPSS, Version 24, IBM). Normality assumptions for parametric tests were all met with the Shapiro-Wilk test. Repeated measures ANOVA with Bonferroni adjustments for the multiple comparisons were used to determine the kinematic adjustments, hindfoot eversion, midfoot and forefoot abduction, navicular drop (i.e. lowering of the medial longitudinal arch) and first MTPJ abduction between natural double leg up-right posture and the first position conditions. Stepwise multiple linear regression analyses were used to predict the combination of kinematic strategies: hindfoot eversion, midfoot and forefoot abduction, and navicular drop (i.e. lowering of the medial longitudinal arch), employed to achieve foot abduction in each first position condition. A Pearson’s correlation analysis was used to determine relationships between hindfoot eversion during each first position condition with the knee joint transverse plane measurements from the passive and active external tibiofemoral rotation trials. A probability (*p*) value of < 0.05 was used to determine significance for all the statistical tests performed. Retrospective power calculations were conducted using G*Power (v.3.0.10) [44] for the stepwise multiple linear regression analyses and the Pearson’s correlation analysis.

## Results

**Fig. 6:**
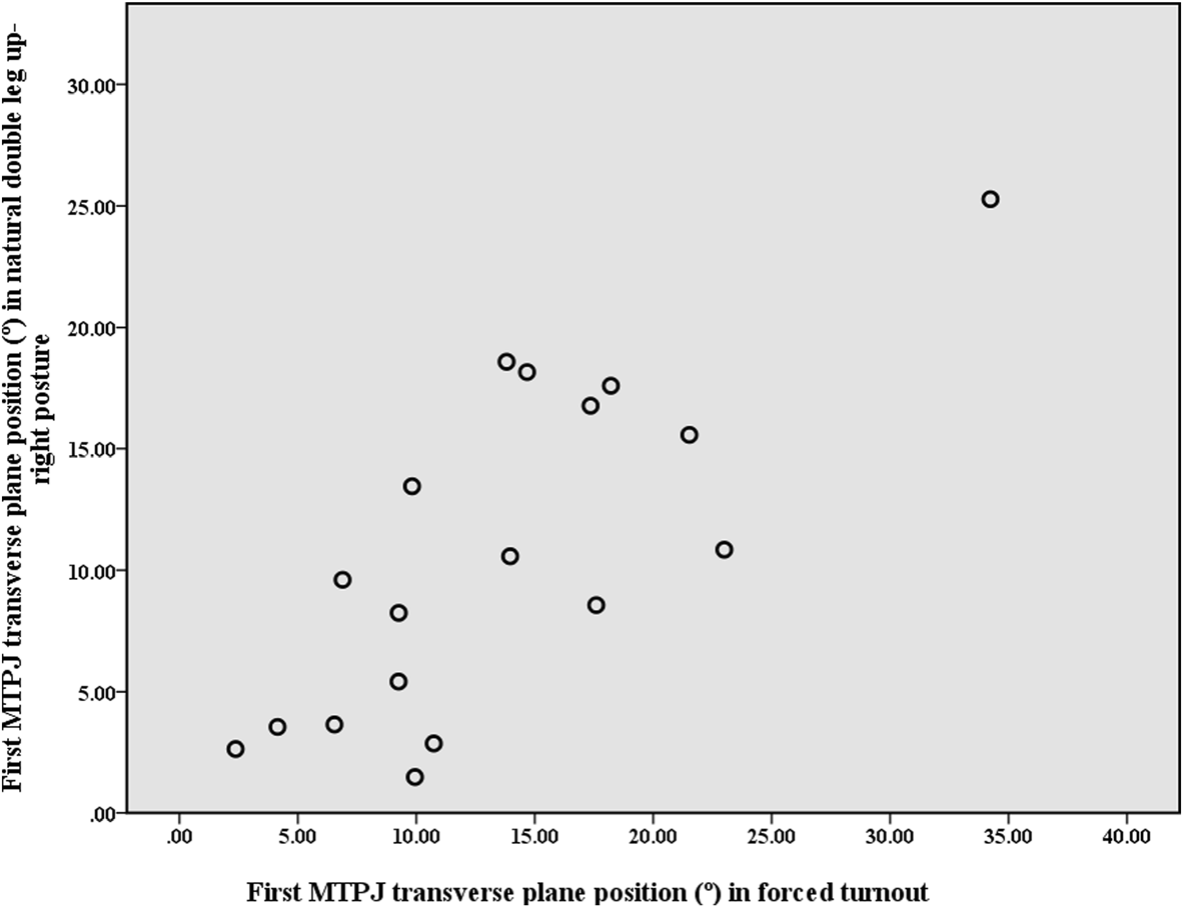
Relationship between first MTPJ transverse plane position (°) in natural double leg up-right posture and in forced turnout (N = 18). *Note:* An abducted first MTPJ is a positive value, and these values are classified as normal (< 15°), ‘mild’ (15–20°), ‘moderate’ (21–39°), and ‘severe’ (≥ 40°) in keeping with Piqué-Vidal & Vila [33]

Seventeen dancers were right limb dominant and one was left limb dominant according to the Waterloo Footedness Questionnaire [34]. All repeated measures ANOVA with Bonferroni adjustments for multiple comparisons revealed significant effects when incorporating a Greenhouse-Geisser correction for hindfoot eversion (*F*_2, 32_ = 74.96, *p* < 0.001), midfoot abduction (*F*_2, 33_ = 39.08, *p* < 0.001), forefoot abduction (*F*_2, 35_ = 4.23, *p* = 0.021), navicular drop (*F*_2, 35_ = 81.51, *p* < 0.001) and first MTPJ abduction (*F*_3, 51_ = 4.07, *p* = 0.011) (Table 2). There were, however, no significant differences between the natural double leg up-right posture and the three first position conditions for the first MTPJ transverse plane position. Interestingly, of the 18 dancers, six dancers had a hallux valgus deformity, five ‘mild’ and one ‘moderate’ (Fig. 6). Thirteen dancers demonstrated an increase in forced turnout, of those three were ‘mild’ and three were ‘moderate’.

**Table 2:** Repeated measures ANOVA for kinematic variables (N = 18)

Kinematic variable^a^	Natural double leg up-right posture	Functional turnout	Forced turnout	Sautés in first position
	Mean (SE) (95CI)	Mean (SE) (95CI)	Mean (SE) (95CI)	Mean (SE) (95CI)
Hindfoot eversion	1.1 (0.8) (− 0.6 to 2.8)	5.7 (0.9)* (3.7 to 7.6)	7.1 (0.9)* ^d^ (5.2 to 9.0)	15.8 (1.6)* (12.4 to 19.1)
Midfoot abduction	2.8 (1.2) (0.2 to 5.4)	5.6 (1.3)* ^c^ (2.8 to 8.4)	6.3 (1.3)* ^e^ (3.5 to 9.2)	7.7 (1.3)* (5.0 to 10.5)
Forefoot abduction	7.6 (1.0) (5.4 to 9.8)	8.4 (1.0) (6.2 to 10.6)	8.8 (1.0)^f^ (6.7 to 10.9)	8.7 (1.1) (6.3 to 11.0)
Navicular drop (mm)^b^		1.6 (0.9) (− 0.3 to 3.5)	1.9 (1.0) (− 0.2 to 4.1)	12.9 (1.2)† (10.4 to 15.5)
First MTPJ abduction	10.7 (1.6) (7.3 to 14.1)	12.0 (1.6) (8.7 to 15.3)	13.5 (1.8) (9.7 to 17.4)	13.1 (1.8) (9.4 to 16.8)

*A significant difference relative to natural double leg up-right posture *p* < 0.001

†A significant difference relative to functional turnout *p* < 0.001

^a^Angle values expressed in degrees unless otherwise signified

^b^A negative value represents an increase in the navicular tuberosity height. A measurement error < 1 mm

^c^This significant result should be interpreted with caution because of the small difference

^d^A significant difference of *p = 0.033* relative to functional turnout. This significant result should be interpreted with caution because of the small difference

^e^A significant difference of *p = 0.012* relative to functional turnout. This significant result should be interpreted with caution because of the small difference

^f^A significant difference of *p = 0.042* relative to natural double leg up-right posture. This significant result should be interpreted with caution because of the small difference

Abbreviations: *SE* standard error; 95CI, 95% confidence interval

**Fig. 7:**
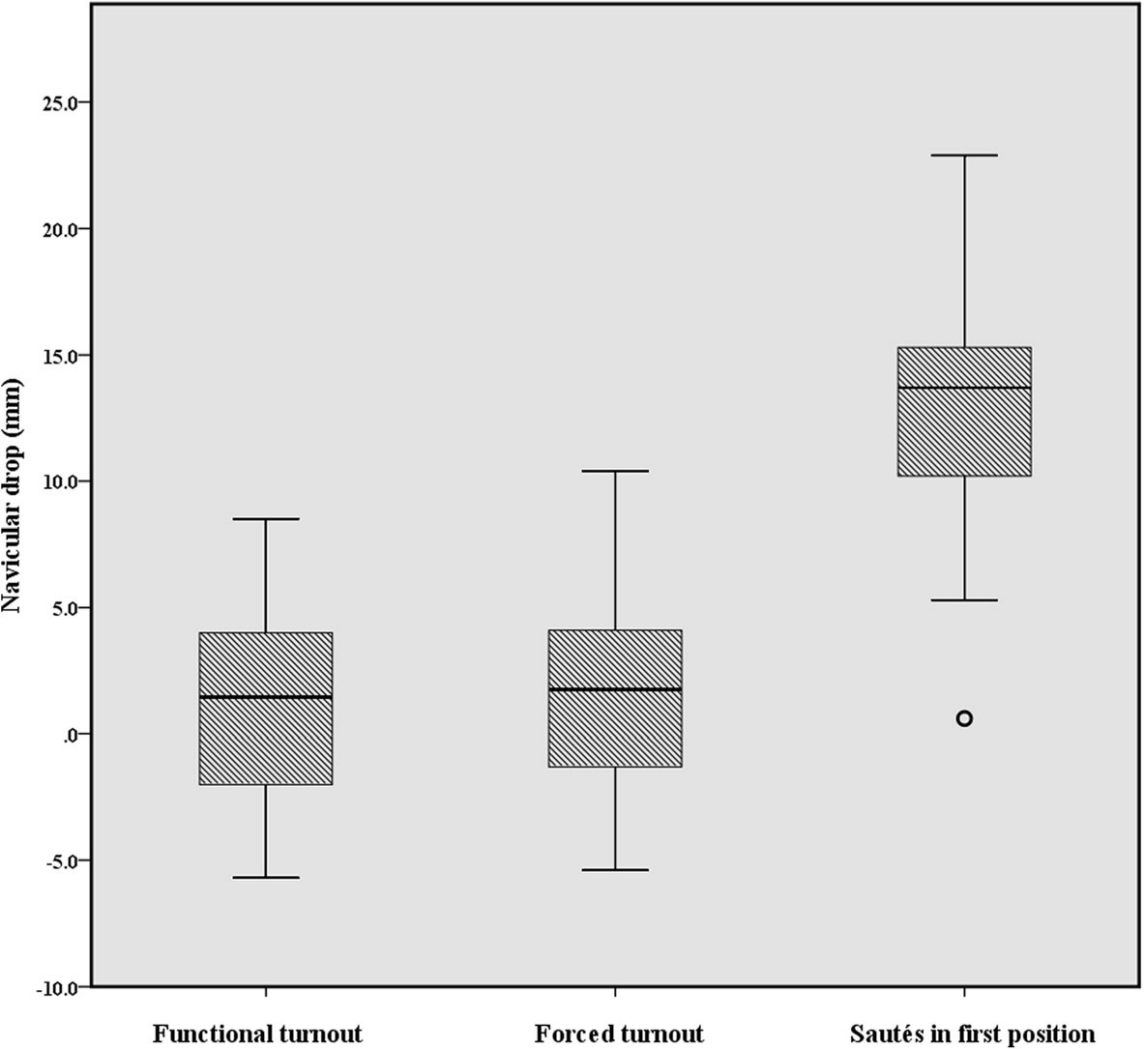
The navicular drop (mm) values across functional turnout, forced turnout and sautés in first position (N = 18). *Note:* Navicular drop was classified into three types of arch height stability according to Gontijo et al. [43]: 1. ‘excellent stabilisation’ navicular drop < 7 mm); 2. ‘stable’ (navicular drop from 7 to 13 mm); 3. ‘unstable’ (navicular drop > 13 mm)

No significant differences were found between navicular drop measurements in functional and forced turnout, i.e. the medial longitudinal arch did not significantly change. All dancers demonstrated either ‘excellent stability’ or ‘stable’ static arch heights in functional and forced turnout (Fig. 7). Seven dancers even demonstrated an increase in navicular height in both standing functional turnout and forced turnout. There was a significant increase in navicular drop during dynamic movements, i.e. the sautés in first position with a drop of 11 mm (*p* < 0.001), with ten dancers exhibiting dynamic arch height ‘instability’.

Multiple regression analyses for foot abduction in functional turnout (*F*_1, 16_ = 19.25.89, *p* < 0.001) revealed hindfoot eversion as positive predictor that could account for 55% variance of foot abduction in functional turnout (*R*^*2*^ = 0.55, Adjusted *R*^*2*^ = 0.52). Retrospective power calculation revealed the model had a power of 0.75; to achieve a power of 0.80, 20 dancers must be recruited.

A similar prediction model was found for forced turnout (*F*_1, 16_ = 21.55, *p* < 0.001) with hindfoot eversion accounting for 57% variance of forced turnout (*R*^*2*^ = 0.57, Adjusted *R*^*2*^ = 0.55). Retrospective power calculation revealed the model had a power of 0.73; to achieve a power of 0.80, 21 dancers must be recruited.

The sautés in first position prediction model (*F*_1, 16_ = 11.761, *p* = 0.003) revealed hindfoot eversion accounted for 42% variance of foot abduction (*R*^*2*^ = 0.42, Adjusted *R*^*2*^ = 0.39) Retrospective power calculation revealed the model had a power of 0.84.

A moderate to strong negative relationship between active external tibiofemoral rotation and hindfoot eversion was found in all three first position conditions (Table 3). Whereas, passive external tibiofemoral rotation was only moderately negatively correlated with hindfoot eversion in all three first position conditions (*r* = − 0.442 to − 0.604) (Table 3). Retrospective power calculation revealed only correlations above 0.58 had a power of 0.80.

**Table 3:** Correlations between the external tibiofemoral rotation clinical measurements and hindfoot eversion (*N* = 18)

		Functional turnout		Forced turnout		Sautés in first position	
		*r*	*p*	*r*	*p*	*r*	*p*
Passive external tibiofemoral rotation	Hindfoot eversion	−0.604	0.008	−0.533	0.023	−0.442	0.066
Active external tibiofemoral rotation	Hindfoot eversion	−0.625	0.006	−0.593	0.009	−0.509	0.031

Abbreviations: *r*, Pearson’s correlation

## Discussion

The primary purpose of this study was to use a dance specific 3D multi-segment foot model to determine the lower leg and foot contributions to turnout that female university-level ballets use to accentuate their turnout. Our results revealed the hindfoot was more everted in forced turnout when compared to functional turnout, although the difference was very small (1.4°, *p* = 0.033) and hence is not meaningful. Therefore, our findings reject our hypothesis. It is important, however to note the hindfoot was 4.6° (*p* < 0.001) more everted in functional turnout compared to natural double leg up-right posture. This suggests hindfoot pronation does play a role in achieving functional turnout, but opens the question of how much is a safe level of pronation? There is a safe range of foot pronation, which facilitates shock absorption and allows for adaption to the ground surface in the gait cycle [16]. There is likely a safe range of hindfoot pronation in turnout, which does not lead to injury. One should also consider the duration and velocity of hindfoot pronation during jump landings which influences the rate at which forces are applied to the foot. Future studies conducting injury surveillance and functional dance screenings using motion analysis should consider range, duration and velocity of hindfoot pronation with the lower extremity in a maximally externally rotated position.

We also surmised a dancer’s foot would undergo forefoot and midfoot abduction and collapse of the medial longitudinal arch in forced turnout compared to functional turnout. We found no change in forefoot abduction angles in functional or forced turnout, but rather an increase in midfoot abduction, although the difference was very small (0.7°, *p* = 0.042) and therefore signifies no meaningful change. Hence, our findings rejected our hypothesis. Midfoot was more abducted (*p* < 0.001) in all three first position conditions compared to natural double leg up-right posture. The midfoot abduction does confirm the unlocking of the midtarsal joint (talocalcaneonavicular and calcaneocuboid joints), which would give the appearance of an abducted forefoot, however the tarsometatarsal joints may not be contributing to a greater abduction angle of the entire foot. Dancers exhibiting greater midfoot transverse mobility, however will expose the medial joint capsule, plantar calcaneonavicular ligament and tibionavicular fibres of the deltoid ligament to increased tensile forces. Repetitive stress on the soft tissue structures could potentially result in further laxity and hinder their function to provide static midfoot stability. However, due care must be taken when comparing the midfoot abduction angle over multiple data collection sessions as the intra and inter-assessor repeatability measures for this angle was poor [28].

Again, contrary to our hypothesis our results demonstrated pre-professional dancer’s arches did not significantly drop when forcing turnout, and even increased in height when assuming functional turnout. The lack of a significant navicular drop in functional turnout was also reported by de Mello Viero et al. [45]. Dancers are educated to activate their arches and to have three points of contact with the ground; the heel, and the first and fifth metatarsal heads, when performing pliés [46]. Pre-professional dancers undergo years of extensive dance education on technique development and strength, which may explain the strong static extrinsic and intrinsic musculature of the foot to maintain good posture in turnout. Although, this strong static musculoskeletal control may not necessarily extend to quick dynamic dance movements. According to Gontijo et al. [43] arch height stability classification, some of the dancers who demonstrated ‘excellent stability’ in both static first positions experienced dynamic ‘instability’ during sautés landings. These dancers demonstrating increased dynamic arch ‘instability’, and therefore, potentially are exposing the posterior tibial muscle to excessive eccentric forces and tensile loads [47]. These mechanical ground reaction forces transferred through the tibia may also increase the likelihood of the dancer developing medial tibial stress syndrome [47]. Eichelberger et al. [48] has recently published a minimal marker-set for measuring navicular drop and drift which could be incorporated into functional dance screenings or even the dance studio, as static measurements of a dancer’s navicular drop provided little indication of dynamic behaviour of the arch height stability. Dance teachers often focus on the arches of a dancer when examining aesthetics. Motion analysis could be used in conjunction with visual and audible cues from the educator to provide positive reinforcement and constructive criticism on their landing technique; instructing the dancer to not collapse through the feet.

Dancers demonstrated no significant increase in the first MTPJ abduction angle when a dancer assumes forced turnout compared to their natural double leg up-right posture. Therefore, our findings reject our hypothesis. Hallux valgus, however, was observed in six of the dancers. The dancer with the most severe deformity was a modern dancer who does not practice en pointe whereas the dancer with the least severe was a classical ballet dancer who trains 20 h a week en pointe. This further supports Steinberg et al. [29] findings in which there is no association between the dance demographics (hours and type of dance practice, en pointe shoe practice) of young dancers with and without hallux valgus. A third of the dancers within this study have hallux valgus, which is only slightly greater than the general female population (aged 18–65, 26.3%) [49], however we believe forcing turnout may be a minor predisposing factor for hallux valgus development, but further investigation is needed with a larger population to provide more clarity. Other predisposing factors suggested for the development of hallux valgus in pre-professional dancers are the anatomical structure of the first MTPJ and genetic factors [29, 50, 51]. Anatomical variation of the shape of the first metatarsal head has been cited as a potential risk factor in the development of hallux valgus, with a round head increasing the risk of developing a hallux valgus deformity compared to a flat head, which resists hallux abduction [52]. Therefore, dancers with a round metatarsal head and forced their turnout may be more at risk of developing of hallux valgus. In addition, the shape of the metatarsal head has been a cited as a risk factor for the reoccurrence of hallux valgus post-surgery [53]. Radiographic examination of the curvature of the first metatarsal head and clinical assessments on first ray dorsal and dorso-medial mobility [51] may provide valuable information in predicting a dancer’s risk of developing hallux valgus deformity.

Hindfoot eversion was the strongest predictor of foot abduction during standing and dynamic first position conditions. Although, retrospective power analysis revealed only the prediction model for the sautés had a power above 0.8. To achieve a power of 0.8 for all the models, a minimum of 21 dancers would be required. Future studies should take this into consideration. Our findings do support our hypothesis that hindfoot eversion is the primary contributor in producing a greater turnout angle during sautés in first position, however more research is required to establish this relationship for the static first position conditions.

Dancers with greater active external tibiofemoral rotation demonstrated a less everted hindfoot during functional and forced turnout postures. Limited hindfoot motion exposes the tibiofemoral joint to excessive external rotation. This supports our findings from earlier studies [3, 12], however the relationship only holds true to active measurements of external tibiofemoral rotation and static first position conditions. Therefore, our findings reject our hypothesis, as we solely stated passive measurements rather than passive and active measurements. These dancers utilising active external tibiofemoral rotation to increase their turnout during barre exercises, resulting in poor knee-foot alignment, may be at greater risk of medial joint capsule and ligaments leading to joint instability. Whereas those with limited active external tibiofemoral rotation may be more prone to Achilles tendinopathies when forcing turnout at the barre. A hyper pronated hindfoot will cause high eccentric forces and torsional stress through the medial Achilles tendon fibres, thereby reducing the shock attenuating capacity of the tendon and ultimately predispose the dancer to micro-tears of the Achilles tendon [54].

### Clinical implications

- Navicular height and/or arch height static measurements do not reliably predict a dancers’ dynamic arch height stability.
- Radiographic assessments of the first MTPJ may assist in determining a dancers’ risk of developing hallux valgus.
- Active measurements of external tibiofemoral rotation can aid clinicians in predicting a dancer’s below-hip compensation mechanism in first position turnout.
- Clinicians and dance educators should monitor the knee-foot alignment of dancers with excessive external tibiofemoral rotation in the interests of preventing knee injuries associated with forced turnout.

## Conclusion

Our findings suggest dancers do pronate, via hindfoot eversion and midfoot abduction in both functional and forced turnout, however, no immediate association was found between forced turnout and first MTPJ abduction. Foot pronation does play a role in achieving turnout. There may be a ‘safe’ range of pronation for turnout postures in dance. Further prospective research on in situ measures of the lower limb in turnout and injury surveillance is required to improve our understanding of the normal and abnormal dance biomechanics.

## Additional file

Additional file 1: The intra- and inter-assessor repeatability of the first MTPJ abduction angle. (DOCX 15 kb)

## Abbreviations

3D: Three-dimensional
95CI: 95% confidence interval
MTPJ: Metatarsophalangeal joint
SD: Standard deviation
SE: Standard error

## References

[1] Ahonen J (2008). Biomechanics of the foot in dance: a literature review. J Dance Med Sci..

[2] Conti S, Wong Y (2001). Foot and ankle injuries in the dancer. J Dance Med Sci..

[3] Carter SL, Duncan R, Weidemann AL, Hopper LS. Lower leg and foot contributions to turnout in female pre-professional dancers: a 3D kinematic analysis. J Sport Sci. 2018:1–9. 10.1080/02640414.2018.1446386 29498315

[4] Wilson GBL (1961). A dictionary of ballet.

[5] Nowacki R, Air ME, Rietveld AB (2012). Hyperpronation in dancers incidence and relation to calcaneal angle. J Dance Med Sci..

[6] Cimelli SN, Curran SA (2012). Influence of turnout on foot posture and its relationship to overuse musculoskeletal injury in professional contemporary dancers. J Am Podiatr Med Assoc.

[7] van Dijk CN, Marti RK (1999). Traumatic, post-traumatic and over-use injuries in ballet: with special emphasis on the foot and ankle. Foot Ankle Surg.

[8] Morton J (2013). The virtuoso foot. Clin Rheumatol.

[9] Liederbach M. Functional evaluative tests for dance: Harkness center for dance injuries. New York, USA: Harkness center for dance injuries; 1994. https://med.nyu.edu/hjd/harkness/sites/default/files/harkness/functional_evaluative_tests_revised.pdf. Accessed 30 Oct 2014.

[10] Gontijo KNS, Candotti CT, Feijó GDS, Ribeiro LP, Loss JF (2017). Dynamic evaluation method of lower limbs joint alignment (MADAAMI) for dancers during the plié. Rev Bras Ciênc Esporte (Impr).

[11] Steinberg N, Hershkovitz I, Peleg S, Dar G, Masharawi Y, Siev-Ner I (2011). Paratenonitis of the foot and ankle in young female dancers. Foot Ankle Int..

[12] Carter SL, Bryant A, Hopper LS (2017). Lower leg and foot contributions to turnout in university-level female ballet dancers: a preliminary investigation. J Am Podiatr Med Assoc.

[13] Redmond AC, Crosbie J, Ouvrier RA (2006). Development and validation of a novel rating system for scoring standing foot posture: the foot posture index. Clin Biomech.

[14] Brody DM (1982). Techniques in the evaluation and treatment of the injured runner. Orthop Clin North Am.

[15] Dicharry JM, Franz JR, Della Croce U, Wilder RP, Riley PO, Kerrigan DC (2009). Differences in static and dynamic measures in evaluation of talonavicular mobility in gait. J Orthop Sports Phys Ther..

[16] Wernick J, Volpe RG, Valmassy RL (1996). Lower extremity function and normal mechanics. Clinical biomechanics of the lower extremities.

[17] Coplan JA (2002). Ballet dancer's turnout and its relationship to self-reported injury. J Orthop Sports Phys Ther..

[18] Jenkins JB, Wyon M, Nevill A (2013). Can turnout measurements be used to predict physiotherapist-reported injury rates in dancers?. Med Probl Perform Art.

[19] Negus V, Hopper D, Briffa K (2005). Associations between turnout and lower extremity injuries in classical ballet dancers. J Orthop Sports Phys Ther.

[20] van Merkensteijn GG, Quin E (2015). Assessment of compensated turnout characteristics and their relationship to injuries in university level modern dancers. J Dance Med Sci..

[21] Khan KM, Brown J, Way S, Vass N, Crichton K, Alexander R (1995). Overuse injuries in classical ballet. Sports Med.

[22] Macintyre J, Joy E (2000). Foot and ankle injuries in dance. Clin Sports Med.

[23] Malkogeorgos A, Mavrovouniotis F, Zaggelidis G, Ciucurel C (2011). Common dance related musculoskeletal injuries. J Phys Educ Sport.

[24] Somogyi DM (2001). Lower leg injuries in dance. J Dance Med Sci.

[25] Kadel N (2014). Foot and ankle problems in dancers. Phys Med Rehabil Clin N Am.

[26] Leardini A, Benedetti MG, Berti L, Bettinelli D, Nativo R, Giannini S (2007). Rear-foot, mid-foot and fore-foot motion during the stance phase of gait. Gait Posture.

[27] Portinaro N, Leardini A, Panou A, Monzani V, Caravaggi P. Modifying the Rizzoli foot model to improve the diagnosis of pes-planus: application to kinematics of feet in teenagers. J Foot Ankle Res. 2014. 10.1186/s13047-014-0057-2. 10.1186/s13047-014-0057-2 PMC4282742 25558289

[28] Carter SL, Sato N, Hopper LS (2017). Kinematic repeatability of a multi-segment foot model for dance. Sports Biomech.

[29] Steinberg N, Siev-Ner I, Zeev A, Dar G (2015). The association between hallux valgus and proximal joint alignment in young female dancers. Int J Sports Med.

[30] Davenport KL, Simmel L, Kadel N (2014). Hallux valgus in dancers. J Dance Med Sci..

[31] Einarsdόttir H, Troell S, Wykman A (1995). Hallux valgus in ballet dancers: a myth?. Foot Ankle Int.

[32] van Dijk CN, Lim LS, Poortman A, Strübbe EH, Marti RK (1995). Degenerative joint disease in female ballet dancers. Am J Sports Med.

[33] Piqué-Vidal C, Vila J. A geometric analysis of hallux valgus: correlation with clinical assessment of severity. J Foot Ankle Res. 2009. 10.1186/1757-1146-2-15. 10.1186/1757-1146-2-15 PMC2694774 19442286

[34] Elias LJ, Bryden MP, Bulman-Fleming MB (1998). Footedness is a better predictor than is handedness of emotional lateralization. Neuropsychologia.

[35] Besier TF, Sturnieks DL, Alderson JA, Lloyd DG (2003). Repeatability of gait data using a functional hip joint Centre and a mean helical knee axis. J Biomech.

[36] Ehrig RM, Taylor WR, Duda GN, Heller MO (2007). A survey of formal methods for determining functional joint axes. J Biomech.

[37] Ehrig RM, Taylor WR, Duda GN, Heller MO (2006). A survey of formal methods for determining the Centre of rotation of ball joints. J Biomech.

[38] Winter DA (2009). Biomechanics and motor control of human movement.

[39] Cappozzo A, Catani F, Della Croce U, Leardini A (1995). Position and orientation in space of bones during movement: anatomical frame definition and determination. Clin Biomech.

[40] Wu G, Cavanagh PR (1995). ISB recommendations for standardization in the reporting of kinematic data. J Biomech.

[41] Wu G, Siegler S, Allard P, Kirtley C, Leardini A, Rosenbaum D (2002). ISB recommendation on definitions of joint coordinate system of various joints for the reporting of human joint motion—part I: ankle, hip, and spine. J Biomech.

[42] Portney L, Watkins M (2015). Foundations of clinical research: applications to practice.

[43] Gontijo KN, Candotti CT, Feijó Gdos S, Ribeiro LP, Loss JF (2015). Kinematic evaluation of the classical ballet step "plié". J Dance Med Sci..

[44] Faul F, Erdfelder E, Lang A-G, Buchner A (2007). G* power 3: a flexible statistical power analysis program for the social, behavioral, and biomedical sciences. Behav Res Methods.

[45] de Mello Viero CC, Kessler LP, Pinto C, Gontijo KNS, da Rosa RG, Kleiner A (2017). Height of the medial longitudinal arch during classical ballet steps. J Dance Med Sci..

[46] Franklin EN. Dance imagery for technique and performance. 2nd ed. Champaign, IL, USA: Human Kinetics; 2013.

[47] Rathleff MS, Kelly LA, Christensen FB, Simonsen OH, Kaalund S, Laessoe U (2012). Dynamic midfoot kinematics in subjects with medial tibial stress syndrome. J Am Podiatr Med Assoc.

[48] Eichelberger P, Blasimann A, Lutz N, Krause F, Baur H. A minimal markerset for three-dimensional foot function assessment: measuring navicular drop and drift under dynamic conditions. J Foot Ankle Res. 2018. 10.1186/s13047-018-0257-2. 10.1186/s13047-018-0257-2 PMC5907216 29713385

[49] Nix S, Smith M, Vicenzino B. Prevalence of hallux valgus in the general population: a systematic review and meta-analysis. J Foot Ankle Res. 2010. 10.1186/1757-1146-3-21. 10.1186/1757-1146-3-21 PMC2955707 20868524

[50] Steinberg N, Hershkovitz I, Peleg S, Dar G, Masharawi Y, Zeev A (2013). Morphological characteristics of the young scoliotic dancer. Phys Ther Sport.

[51] Biz C, Favero L, Stecco C, Aldegheri R (2013). Hypermobility of the first ray in ballet dancer. Muscles Ligaments Tendons J.

[52] Karasick D, Wapner KL (1990). Hallux valgus deformity: preoperative radiologic assessment. Am J Roentgenol.

[53] Okuda R, Kinoshita M, Yasuda T, Jotoku T, Kitano N, Shima H (2007). The shape of the lateral edge of the first metatarsal head as a risk factor for recurrence of hallux valgus. J Bone Joint Surg Am.

[54] Schepsis AA, Jones H, Haas AL (2002). Achilles tendon disorders in athletes. Am J Sports Med.

[55] Gans A (1985). The relationship of heel contact in ascent and descent from jumps to the incidence of shin splints in ballet dancers. Phys Ther.

[56] Orishimo KF, Kremenic IJ, Pappas E, Hagins M, Liederbach M (2009). Comparison of landing biomechanics between male and female professional dancers. Am J Sports Med.

