# Supplementary material for: An analysis of the foot in turnout using a dance specific 3D multi-segment foot model
Source: J Foot Ankle Res. 2019 Feb 4;12:10. doi: 10.1186/s13047-019-0318-1 (PMC6360724; doi:10.1186/s13047-019-0318-1)
Supplement: Supplementary file 1 — The intra- and inter-assessor repeatability of the first MTPJ abduction angle. (DOCX 15 kb) [file 13047_2019_318_MOESM1_ESM.docx]

Repeatability of the first MTPJ abduction angle

The intra- and inter-assessor repeatability of the first MTPJ abduction angle was determined using legacy data from Carter et al. (1).

We ran the VICON Bodylanguage script stated below through the original repeatability datasets, which included four data collection sessions. Each session consisted of three repetitions of the following static trials: parallel stance, turnout stance, turnout pliés and turnout rise. The turnout rise trials were not included because of the coupling between the sagittal and transverse measurements, when the first MTPJ reaches extreme dorsiflexion, therefore reducing the accuracy of measuring first MTPJ abduction angle.

Hallux abduction angles were extracted from the parallel stance, turnout stance and turnout plié trials, using MATLAB (custom software written using MATLAB, MathWorks Inc., USA). An average of three trials for each task was determined using MATLAB.

Intra and inter-assessor repeatability of the first MTPJ abduction angles were calculated Intra-assessor, the difference between session one and two; Inter-assessor: the difference between the two assessors for session one. All statistical analyses were calculated using SPSS for Windows Version 21 (SPSS Inc., Chicago, Illinois, USA). The static ICC values were calculated using the angular (°) difference between two sessions using the mean variables measured during turnout plié, turnout stance and parallel stance. The intra- and inter-assessor values for first MTPJ abduction angles demonstrated excellent repeatability with intra-class correlation coefficient values ranging between 0.886 and 0.888.

1. Carter SL, Sato N, Hopper LS. Kinematic repeatability of a multi-segment foot model for dance. Sports Biomechanics. 2017a;17(1):48-66.
